# Diagnostic and prognostic utility of bone morphogenetic protein 10 in acute dyspnea: a cohort study

**DOI:** 10.1007/s00392-024-02584-2

**Published:** 2024-12-11

**Authors:** Justas Simonavicius, Desiree Wussler, Maria Belkin, Karoline Luening, Pedro Lopez-Ayala, Ivo Strebel, Samyut Shrestha, Albina Nowak, Eleni Michou, Androniki Papachristou, Codruta Popescu, Nikola Kozhuharov, Zaid Sabti, Zora Reiffer, Elisa Hennings, Tobias Zimmermann, Matthias Diebold, Tobias Breidthardt, Christian Mueller

**Affiliations:** 1 Cardiovascular Research Institute Basel (CRIB), University Hospital Basel, University of Basel, Petersgraben 4, 4031 Basel, Switzerland; 2 Department of Hematology, University Hospital Basel, Basel, Switzerland; 3 GREAT Network, Rome, Italy; 4 Department of Cardiology, University Hospital Basel, Petersgraben 4, 4031 Basel, Switzerland; 5 Department of Endocrinology and Clinical Nutrition, University Hospital Zurich, Zurich, Switzerland; 6 Division of Internal Medicine, University Psychiatry Clinic Zurich, Zurich, Switzerland; 7 Department of Internal Medicine, University Hospital Basel, Basel, Switzerland; 8 Department of Cardiology, Inselspital, Bern University Hospital, Bern, Switzerland

**Keywords:** BMP10, Diagnosis, Death, Acute heart failure, Atrial fibrillation

## Abstract

**Background and aim:**

The possible clinical utility of Bone Morphogenetic Protein 10 (BMP10), a novel atrial-specific biomarker, is incompletely understood. We aimed to test the hypothesis that BMP10 has high diagnostic and prognostic accuracy in patients presenting with acute dyspnea.

**Methods and results:**

In a multicenter diagnostic study, BMP10, high-sensitivity cardiac troponin T (hs-cTnT), and N-terminal pro-B-type natriuretic peptide (NT-proBNP) concentrations were determined in patients presenting with acute dyspnea to the emergency department. The final diagnosis was centrally adjudicated by two independent cardiologists blinded to BMP10. Diagnostic accuracy for acute heart failure (AHF) was quantified using the area under the receiver operating characteristic curve (AUC). 720-day all-cause mortality and the composite of all-cause mortality or AHF rehospitalization were prognostic endpoints. Among 933 consecutive patients, 54% were adjudicated to have AHF. Patients with AHF had higher BMP10 concentrations (median 3.34 [IQR 2.55–4.35] ng/mL) compared to patients with other causes of acute dyspnea (2.04 [1.74–2.45] ng/mL, *p* < 0.001). The AUC of BMP10 was 0.85 (95%CI, 0.82–0.87), versus 0.79 (95%CI, 0.76–0.82, *p* < 0.001) for hs-cTnT and 0.91 (95%CI, 0.90–0.93, *p* < 0.001) for NT-proBNP. The combination of BMP10 with NT-proBNP (AUC 0.92, 95%CI, 0.90–0.94) did not significantly increase the AUC versus NT-proBNP alone. BMP10 was a powerful predictor of death and AHF rehospitalization, but did not provide incremental value to models including NT-proBNP.

**Conclusion:**

BMP10 had a high diagnostic accuracy for AHF and high prognostic accuracy for death and AHF rehospitalization. However, it did not provide relevant incremental value to the current gold standard NT-proBNP.

**Supplementary Information:**

The online version contains supplementary material available at https://doi.org/10.1007/s00392-024-02584-2.

## Introduction

Acute dyspnea constitutes a significant proportion of emergency department (ED) visits, accounting for approximately 14 million people seeking emergency care in Europe and North America alone each year. A substantial majority of these patients, about two-thirds, necessitate further in-hospital management, placing a huge burden on healthcare systems worldwide [1]. Unfortunately, the early detection of the underlying cause of acute dyspnea including acute heart failure (AHF), obstructive pulmonary disease, pneumonia, pulmonary embolism, and early risk stratification is challenging in many patients. This is a clinical dilemma as early cause-specific therapy, and risk-adopted disposition and management are mandatory to avoid the dismal outcome associated with many of these life-threatening disorders if detected late [1, 2].

Currently, natriuretic peptides, specifically B-Type Natriuretic Peptide (BNP) and N-terminal-pro-B-type natriuretic peptide (NT-proBNP), are recommended by both American and European clinical practice guidelines as aids in the early rule-in or rule-out of AHF [3, 4]. However, the use of BNP and NT-proBNP as first-line diagnostic modalities varies among centers and is often low [5], particularly in Europe, and their diagnostic accuracy is limited in certain clinical scenarios, such as atrial fibrillation [6, 7].

Recently, bone morphogenetic protein 10 (BMP10) has been identified as an atrial-specific circulating biomarker in postnatal individuals [8]. BMP10 is a biologically active protein belonging to the large transforming growth factor (TGF-ß) family that binds to activin receptor-like kinase 1 receptors expressed on endothelial cells, in turn playing a key regulatory role in vascular biology [9]. The transcription factor myocardin regulates BMP10 expression in the heart [10]. BMP10 binding to its receptors initiates a cascade of dose-dependent phosphorylation reactions, forming part of a complex intracellular signaling process [11]. BMP10-knockout mice do not survive in utero and display impaired cardiac trabeculation, [9] whereas a genetically mediated loss-of-function variant in the BMP10 gene has been linked to pulmonary arterial hypertension in humans [12], underscoring its role in pulmonary hemodynamics. Under pathological conditions, such as high-salt diet-induced hypertension in rats and hyperglycemia, BMP10 expression is significantly upregulated, highlighting its pivotal role in maintaining cardiac homeostasis [13, 14].

We recently demonstrated a strong relationship between both BMP10 and NT-proBNP with mean pulmonary artery pressure. However, only BMP10 was associated with elevated pulmonary vascular resistance and not pulmonary wedge pressure, suggesting that it may uniquely reflect complementary aspects of hemodynamic states [15]. Moreover, BMP10 is strongly associated with the presence of atrial fibrillation as well as its complications [16–19], possibly complementing the established risk prediction tools, such as NT-proBNP [17, 19]. Given the pathophysiological atrioventricular interplay and potential complimentary roles, we hypothesized that BMP10 may have diagnostic and prognostic utility in patients presenting with acute dyspnea to the ED. Specifically, we hypothesized that the atrial-specificity of BMP10 would result in incremental diagnostic value for AHF to NT-proBNP, which is released mainly from both ventricles. We aimed to test these hypotheses in a prospective diagnostic study with central adjudication of the cause of acute dyspnea and long-term follow-up.

## Methods

### Design and study population

**Fig. 1:**
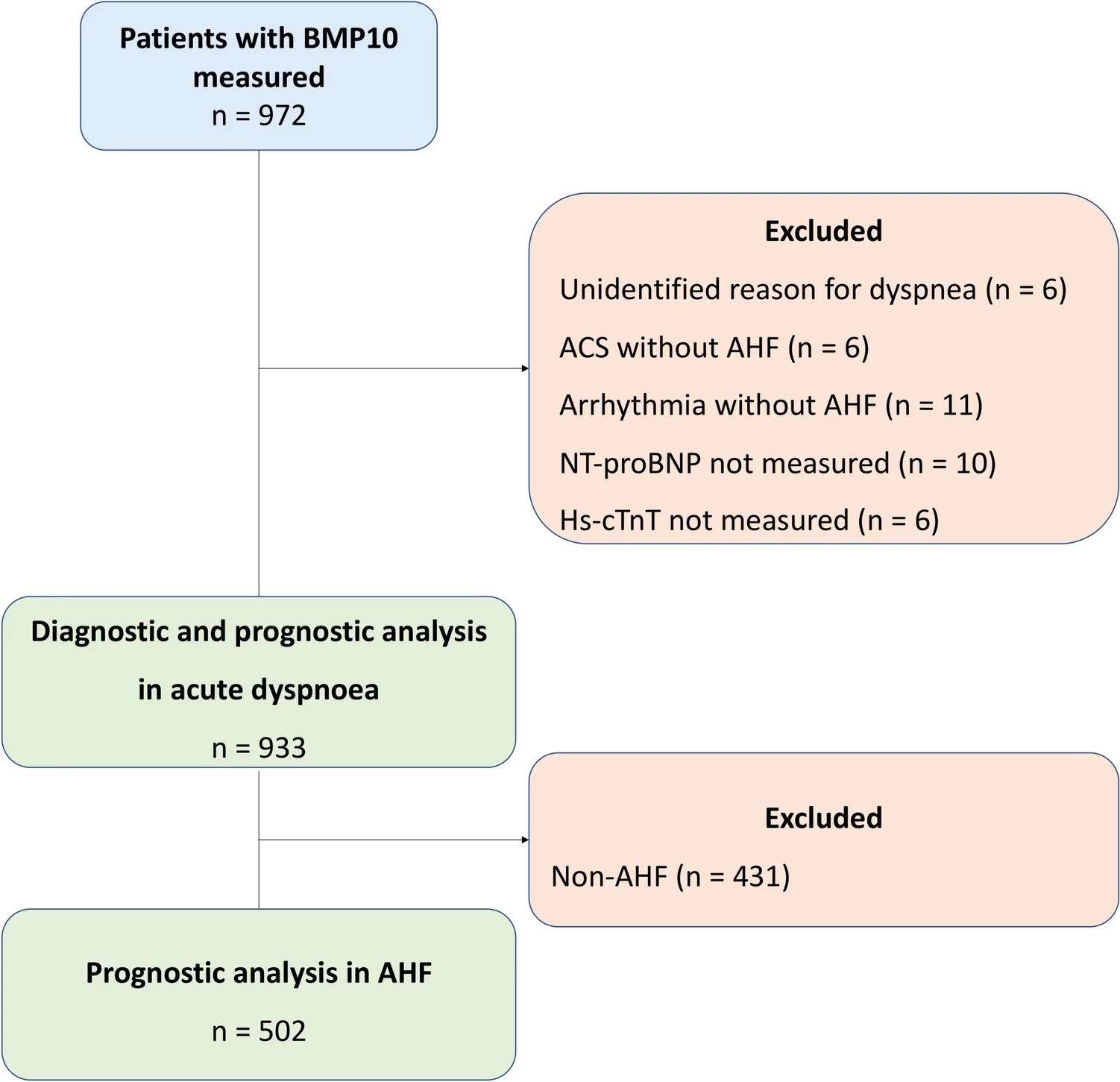
Flowchart of the study population. *ACS* acute coronary syndrome, *AHF* acute heart failure, *BMP10* Bone Morphogenetic Protein 10, *Hs-cTnT* high-sensitivity cardiac troponin T, *NT-proBNP* N-terminal pro-B-type natriuretic peptide

This is a secondary analysis within the Basics in Acute Shortness of Breath EvaLuation (BASEL V) study, a prospective, multicenter diagnostic cohort study aiming to advance the early management of patients with acute dyspnea (ClinicalTrials.gov, NCT01831115) [20–22]. Adult patients who presented with acute dyspnea to the EDs of two University Hospitals in Switzerland (Basel and Zürich) were enrolled. Patients were enrolled irrespective of their renal function at ED presentation, but those with terminal kidney failure on chronic dialysis were excluded. Additionally, patients with an unclear final diagnosis, even after central adjudication, or those who were adjudicated as having cardiac dyspnea due to acute coronary syndrome or arrhythmia without any other evidence of AHF, were also excluded. For this analysis, patients were eligible if they had BMP10, NT-proBNP, and high-sensitivity cardiac troponin T (hs-cTnT) measured from study blood samples taken upon ED presentation (Fig. 1).

This study was carried out according to the principles of the Declaration of Helsinki and was approved by the local ethics committees. All patients provided their written informed consents.

### Adjudication of the final diagnosis

Two independent cardiologists, with access to comprehensive patient information including clinical history, physical examination, 12-lead electrocardiogram, laboratory findings (including BNP or NT-proBNP concentrations), chest X-ray, echocardiography, lung function testing, computed tomography, response to therapy, and 360-day follow-up, centrally adjudicated the main cause of acute dyspnea according to current clinical practice guidelines [20–22]. BMP10 measurements were not available for the central adjudication of the final diagnosis. In cases where there was a disagreement regarding the diagnosis, a third cardiologist was consulted to review and make a joint adjudication decision. Hypertensive-AHF was defined as AHF resulting from a hypertensive state without evident pulmonary edema, whereas pulmonary edema was defined as a clinical syndrome of pulmonary volume overload, with or without underlying hypertension.

### Patient follow-up

Follow-up took place at specific intervals, 90 days, 1 year, and 2 years, through either telephone calls or written communication by trained researchers. The follow-up team was not aware of the patients’ BMP10 plasma concentrations. During the follow-up period, if there were any potential medical events, such as AHF hospitalization or all-cause death, additional information was gathered from various sources, including hospital’s and family physician’s records, or the national death registry.

### Biochemical measurements

At ED presentation, blood samples were collected using potassium ethylene-diamine-tetra-acetic acid. Following centrifugation, the samples were promptly frozen at − 80 °C and stored in a centralized biobank until they were assayed in a blinded manner at a dedicated core laboratory. For the measurement of BMP10 concentrations, a non-commercial, robust prototype electrochemiluminescence immunoassay applying monoclonal antibodies specifically developed against BMP10 (lower detection limit = 0.003 ng/mL, functional sensitivity lower limit of quantification = 0.012 ng/mL) was utilized by means of a Roche cobas e601 analyzer by laboratory personnel blinded to clinical information. [17, 19] Previously performed run‐control measurements resulted in a coefficient of variation of 2.35% (mean 1.38 ng/mL) [19].

For BMP10, stability of the measurements in biological plasma samples with a routine of stress testing conditions had recently been demonstrated as assumed variances of monitored storage conditions are expected to be less critical on sample stability [19]. The test covered incubation of plasma samples stored after thawing from − 80 °C at room temperature for 1 as well as 2 days of 3 independent samples, resulted in recovery of 104% each. Application of freeze/thaw cycles to 10 independent plasma samples revealed high stability for a single cycle (− 80 °C/room temperature/ − 80 °C) of 101% as well as for 3 cycles resulting in 103% [19]. NT-proBNP concentrations were measured using the Elecsys NT-proBNP II assay. hs-cTnT concentrations were determined using the Elecsys hs-cTnT assay (all Roche Diagnostics AG, Rotkreuz, Switzerland). Mid-regional pro-adrenomedullin was measured using automated sandwich chemiluminescence immunoassay on the KRYPTOR system (B·R·A·H·M·S AG, Hennigsdorf/Berlin, Germany), whereas bio-adrenomedullin was measured using a double-monoclonal antibody sandwich immunoassay (Sphingotec GmbH, Hennigsdorf, Germany) as described elsewhere [23]. Estimated glomerular filtration rate (eGFR) was calculated using the Chronic Kidney Disease Epidemiology Collaboration (CKD-EPI) equation [24].

### Echocardiography

Echocardiographic data were extracted from routine medical records without any study-specific echocardiography protocol. The local echocardiography studies adhered to the established local protocols that were in line with the guidelines for chamber quantification. [25] Echocardiographic examinations were performed using high-end ultrasound systems, including (Philips EPIQ and GE Vivid], equipped with phased-array transducers. All echocardiographic examinations were conducted by experienced sonographers and cardiologists with specialized training in echocardiography.

### Outcome measures

#### Diagnostic analysis

The diagnostic accuracy for AHF was quantified by the area under the receiver operating characteristic curve (AUC) as the primary diagnostic outcome measure.

#### Prognostic analysis

All-cause mortality within 720 days was chosen as a primary prognostic outcome, the composite of all-cause mortality or hospitalization for AHF the secondary outcome.

### Statistical analysis

Continuous variables are presented as median and inter-quartile range (IQR) and categorical variables as frequencies and valid percentages. Mann–Whitney U test was used to compare two independent continuous variables, while chi-square test was used to compare categorical variables. The monotonic associations between BMP10 and biomarkers quantifying different pathophysiological processes, as well as echocardiographic parameters, were assessed using Spearman correlation coefficients. The diagnostic performance of BMP10 for diagnosing AHF, as well as AHF triggered by specific factors, such as arrhythmia, ischemia, and infection, was quantified by the area under the receiver operating characteristic curve (AUC). DeLong’s method was used to assess the statistical significance of differences between AUCs. Kaplan–Meier curves stratified by BMP10 quartiles were used to visualize patient outcomes. Log-rank test was used to assess differences between quartiles. To assess the added value of BMP10, multivariable Cox proportional hazard models were fitted. Covariates were selected according to literature [26]. Restricted cubic splines were used to model potential non-linear relationships between circulating biomarkers, including BMP10, NT-proBNP, urea, and the outcome variable. Multivariable Cox models were then used for the computation of outcome probabilities (risk). The added value was reported as fraction of new information, which is the fraction of post-test risk explained by BMP10. The post-test risk is derived from the nested multivariable model with BMP10, whereas the pre-test risk is derived from the nested model without BMP10. For this section of the analysis, adjustments were made for the same variables that were incorporated into the Cox proportional hazards models. Statistical analyses were conducted using SPSS/PC Software Package (version 28.0), MedCalc online tool (MedCalc Software Ltd), and R Statistical Software (version 4.2.2).

## Results

### Patient characteristics and central adjudication

Among 972 consecutively enrolled patients with BMP10 concentrations available, 933 patients (96%) met the criteria for inclusion (Fig. 1). The median age of the patients was 76 (64–83) years, 45% were women, about a third had a history of heart failure, and 30% had atrial fibrillation (Table 1).

**Table 1:** Baseline characteristics of the patients presenting to the emergency department with acute dyspnea stratified by the presence or absence of acute heart failure

	All patients (n-933)	AHF (n-502)	Non-AHF (n-431)	*p*-value
Demographics				
Age (years)	76 (64–83)	80 (70–85)	69 (56–79)	< 0.001
Female sex	416 (45%)	210 (42%)	206 (48%)	0.075
Weight (kg)	75 (63–88)	76 (65–88)	75 (61–88)	0.110
BMI (kg/m^2^)	26.1 (22.7–30.3)	26.4 (23.4–30.3)	25.7 (21.9–30.1)	0.015
Clinical parameters at presentation				
SBP (mmHg)	135 (119–154)	135 (117–155)	135 (120–152)	0.906
Heart rate (bpm)	90 (75–106)	87 (72–105)	93 (78–106)	0.007
Respiratory rate (breaths/minute)	22 (17–28)	22 (18–28)	22 (17–28)	0.915
Pulse oximetry (%)	96 (92–98)	96 (93–98)	96 (92–99)	0.705
Temperature (°C)	37.3 (36.8–37.7)	37.1 (36.6–37.6)	37.4 (37.0–37.9)	< 0.001
Medical history				
Hypertension	667 (72%)	424 (85%)	243 (56%)	< 0.001
Dyslipidemia	448 (48%)	289 (58%)	159 (37%)	< 0.001
CAD	342 (37%)	251 (50%)	91 (21%)	< 0.001
Prior MI	202 (22%)	154 (31%)	48 (11%)	< 0.001
Prior AHF	319 (34%)	275 (55%)	44 (10%)	< 0.001
Atrial fibrillation	284 (30%)	244 (49%)	40 (9%)	< 0.001
CKD	270 (29%)	206 (41%)	64 (15%)	< 0.001
PAD	106 (11%)	74 (15%)	32 (7%)	< 0.001
Stroke	114 (12%)	77 (15%)	37 (9%)	0.002
COPD/Asthma	322 (35%)	125 (25%)	197 (46%)	< 0.001
Medication at presentation				
ACE inhibitors	298 (32%)	204 (41%)	94 (22%)	< 0.001
ARBs	208 (22%)	134 (27%)	74 (17%)	< 0.001
Beta-blockers	454 (49%)	325 (65%)	129 (30%)	< 0.001
Aldosterone antagonists	82 (9%)	65 (13%)	17 (4%)	< 0.001
CCB	176 (19%)	111 (22%)	67 (15%)	0.007
Digoxin	25 (3%)	20 (4%)	5 (1%)	0.008
Diuretics	501 (54%)	356 (71%)	145 (34%)	< 0.001
Echocardiography				
LVEF (%)	50 (39–60)	45 (34–57)	60 (55–60)	< 0.001
Laboratory parameters				
Hemoglobin (g/L)	132 (117–145)	127 (113–140)	139 (126–149)	< 0.001
Hematocrit (%)	39 (35–42)	37 (34–41)	40 (37–43)	< 0.001
Sodium (mmol/L)	139 (136–141)	139 (137–142)	138 (135–140)	< 0.001
Chloride (mmol/L)	100 (97–104)	101 (97–104)	99 (96–102)	< 0.001
Potassium (mmol/L)	4.1 (3.8–4.5)	4.1 (3.8–4.6)	4.0 (3.7–4.3)	< 0.001
Creatinine (µmol/L)	89 (70–122)	102 (80–150)	76 (61–95)	< 0.001
eGFR (ml/min/1.73 m^2^)	65 (43–87)	53 (34–73)	83 (60–97)	< 0.001
Urea (mmol/L)	7.8 (5.4–11.3)	9.7 (6.7–14.2)	6.3 (4.5–8.8)	< 0.001
Albumin (g/L)	35 (32–38)	35 (32–37)	36 (32–39)	< 0.001
NT-proBNP (ng/L)	1708 (273–5624)	4533 (2120–9206)	250 (99–776)	< 0.001
Hs-cTnT (ng/L)	24 (12–50)	37 (21–72)	13 (6–25)	< 0.001

*ACE* angiotensin-converting enzyme, *AHF* acute heart failure, *ARB* angiotensin II receptor blocker, *BMI* body mass index, *CAD* coronary artery disease, *CCB* calcium channel blocker, *CKD* chronic kidney disease, *COPD* chronic obstructive pulmonary disease, *eGFR* estimated glomerular filtration rate, *Hs-cTnT* high-sensitivity cardiac troponin T, *LVEF* left ventricular ejection fraction, *MI* myocardial infarction, *NT-proBNP* N-terminal pro-B-type natriuretic peptide, *PAD* peripheral artery disease, *SBP* systolic blood pressure

Central adjudication revealed AHF as the cause of acute dyspnea in 502 (54%) patients.

### Plasma BMP10 in the study population

**Fig. 2:**
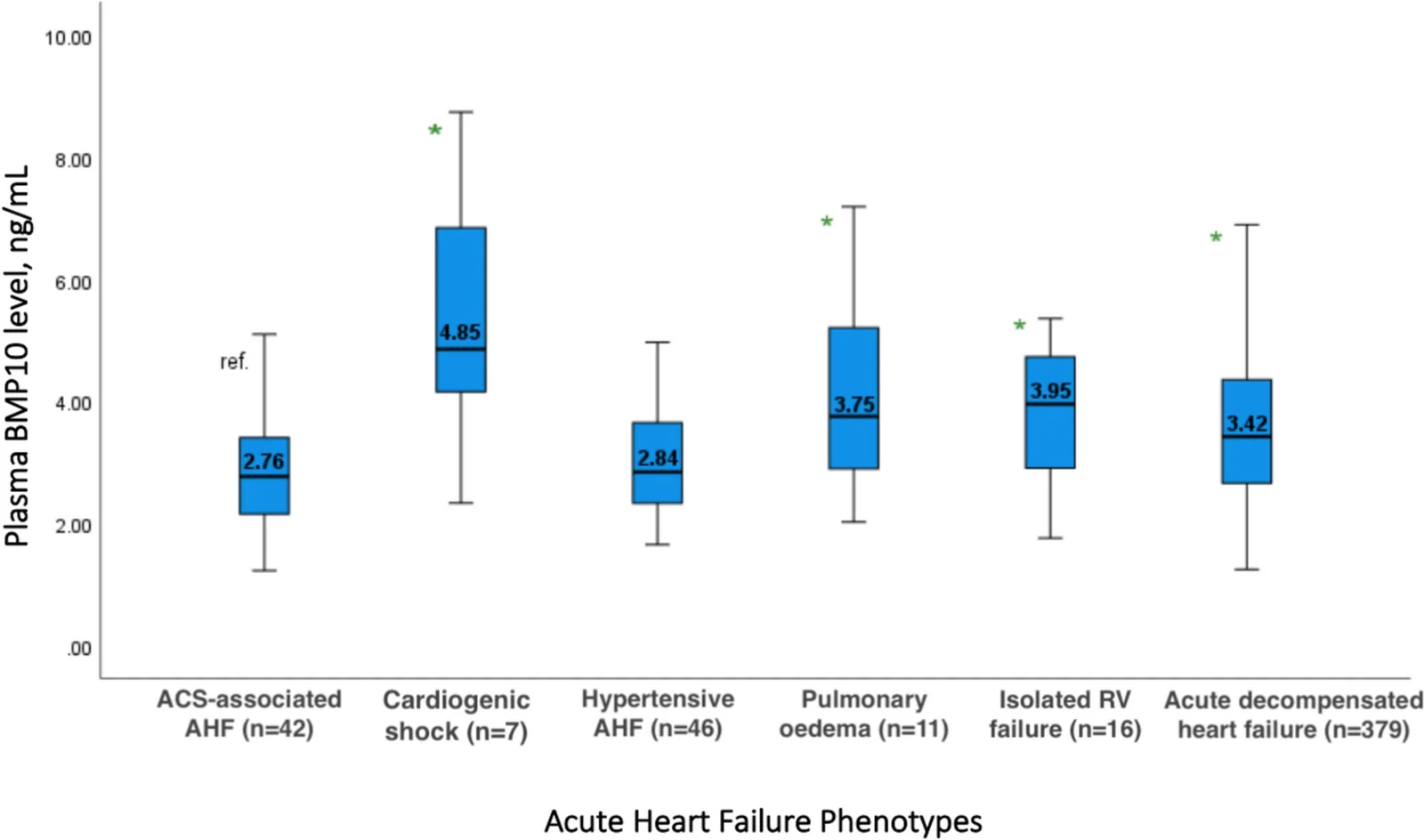
Plasma BMP10 concentration across the different Acute Heart Failure phenotypes. *ACS* acute coronary syndrome, *AHF* acute heart failure, *BMP10* Bone Morphogenetic Protein 10, *RV* right ventricular. *Significant difference between ACS-complicated AHF (reference phenotype) and the corresponding phenotype (*p* < 0.05)

**Fig. 3:**
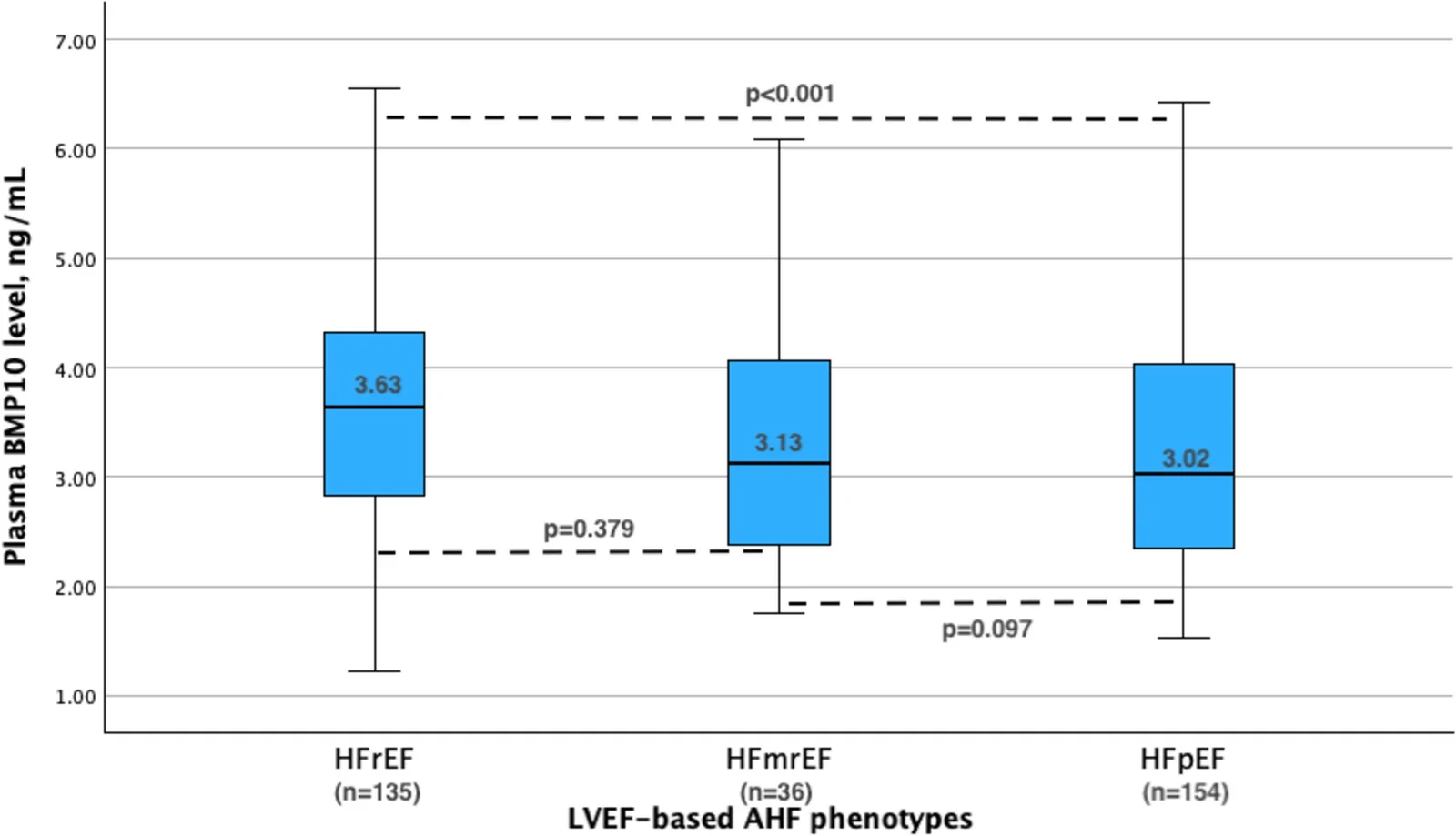
Plasma BMP10 concentration across the different left ventricular ejection fraction phenotypes of Acute Heart Failure. *AHF* acute heart failure, *BMP10* Bone Morphogenetic Protein 10, *HFmrEF* heart failure with mid-range left ventricular ejection fraction, *HFpEF* heart failure with preserved left ventricular ejection fraction, *HFrEF* heart failure with reduced left ventricular ejection fraction, *LFEF* left ventricular ejection fraction

Plasma BMP10 concentration in patients with AHF was higher than in non-AHF patients (3.34 [2.55–4.35] vs 2.04 [1.74–2.45] ng/mL, *p* < 0.001, respectively). Among the different AHF phenotypes, acute coronary syndrome-associated AHF patients had the lowest plasma BMP10 concentration (2.76 [2.15–3.40]), whereas patients with cardiogenic shock had the highest plasma BMP10 concentration (4.85 [4.15–6.85] ng/mL, *p* < 0.001, Fig. 2). Patients with heart failure with reduced ejection fraction (HFrEF) had higher plasma BMP10 concentration as compared to patients with heart failure with preserved ejection fraction (HFpEF, Fig. 3).

BMP10 concentrations were comparable across the different diagnostic categories in the non-AHF group (*p* > 0.05, Supplemental Table 1).

### Diagnostic performance for AHF

**Fig. 4:**
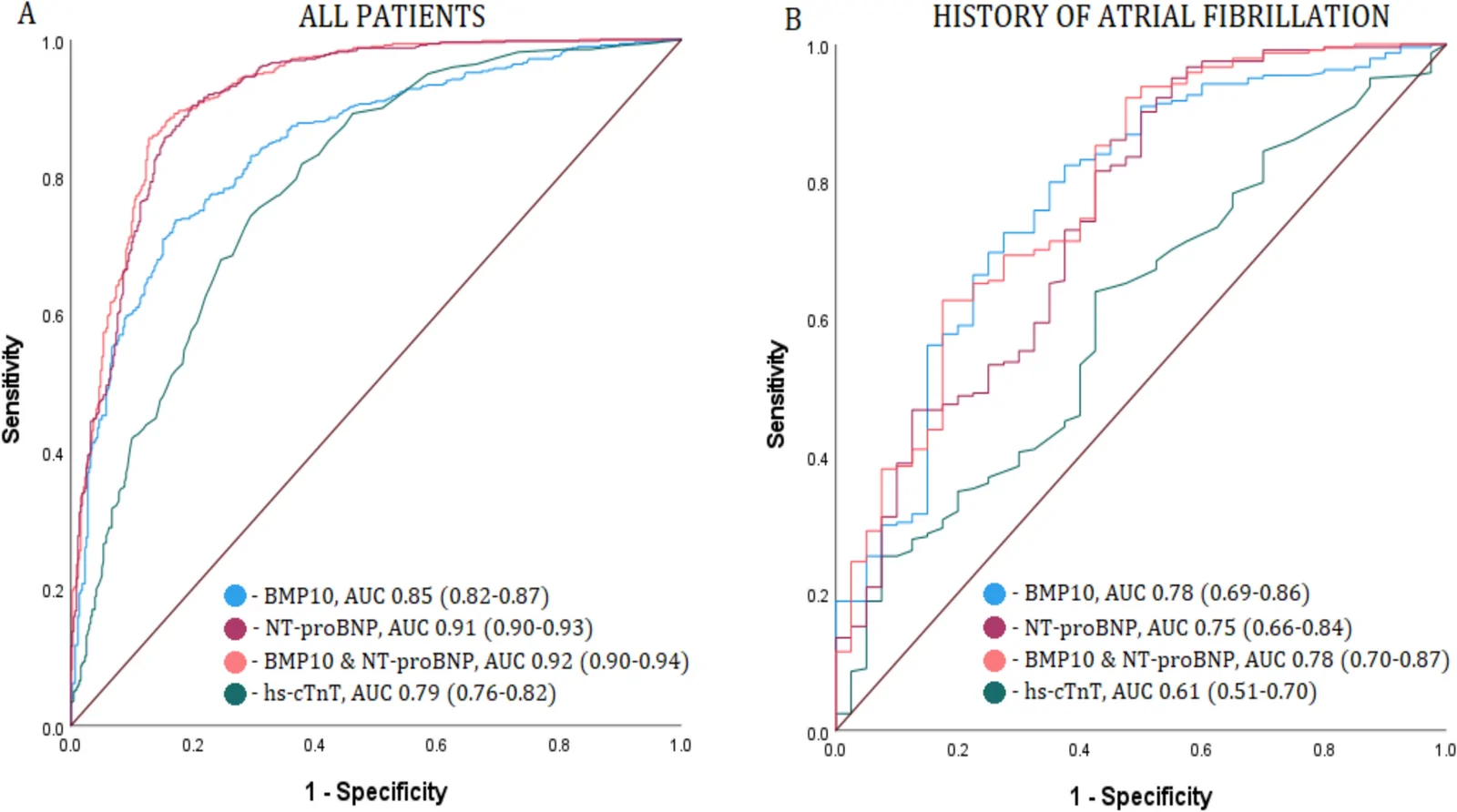
Receiver operating characteristic curves comparison of BMP10 with high-sensitivity cardiac troponin T (hs-cTnT), N-terminal pro-B-type natriuretic peptide (NT-proBNP), and the combination of BMP10 and NT-proBNP for the diagnosis of acute heart failure in all patients (*n* = 933, Fig. 3A), patients with a history of atrial fibrillation (*n* = 284, Fig. 3B)

The diagnostic accuracy of plasma BMP10 levels in identifying AHF, as measured by AUC, was high (0.85, 95% CI 0.82–0.87), significantly higher versus hs-cTnT (0.79, 95% CI 0.76–0.82, *p* for comparison < 0.001), but significantly lower versus NT-proBNP (0.91, 95% CI 0.90–0.93, *p* for comparison < 0.001). When combined with NT-proBNP, BMP10 did not improve the diagnostic accuracy of NT-proBNP alone (AUC 0.92, 95% CI 0.90–0.94, *p* for comparison = 0.64, Fig. 4A). BMP10 did not outperform NT-proBNP among patients with eGFR < 60 ml/min/1.73 m^2^ (Supplemental Fig. 1).

#### Subgroup analysis: atrial fibrillation

Subgroup analysis in patients with a history of atrial fibrillation (*n* = 284) revealed a reduction in the diagnostic accuracy of all three biomarkers (Fig. 4B). AUC for BMP10 was 0.78 (95% CI 0.69–0.86), AUC for NT-proBNP was 0.75 (95% CI 0.66–0.84, *p* for comparison = 0.69), and AUC for hs-cTnT was 0.61 (95% CI 0.51–0.70, *p* for comparison < 0.05). Combining BMP10 and NT-proBNP did not improve the diagnostic accuracy as compared to NT-proBNP alone (AUC 0.78, 95% CI 0.70–0.87, *p* for comparison = 0.58).

### Characterization of plasma BMP10 by its correlation with established blood biomarkers

**Fig. 5:**
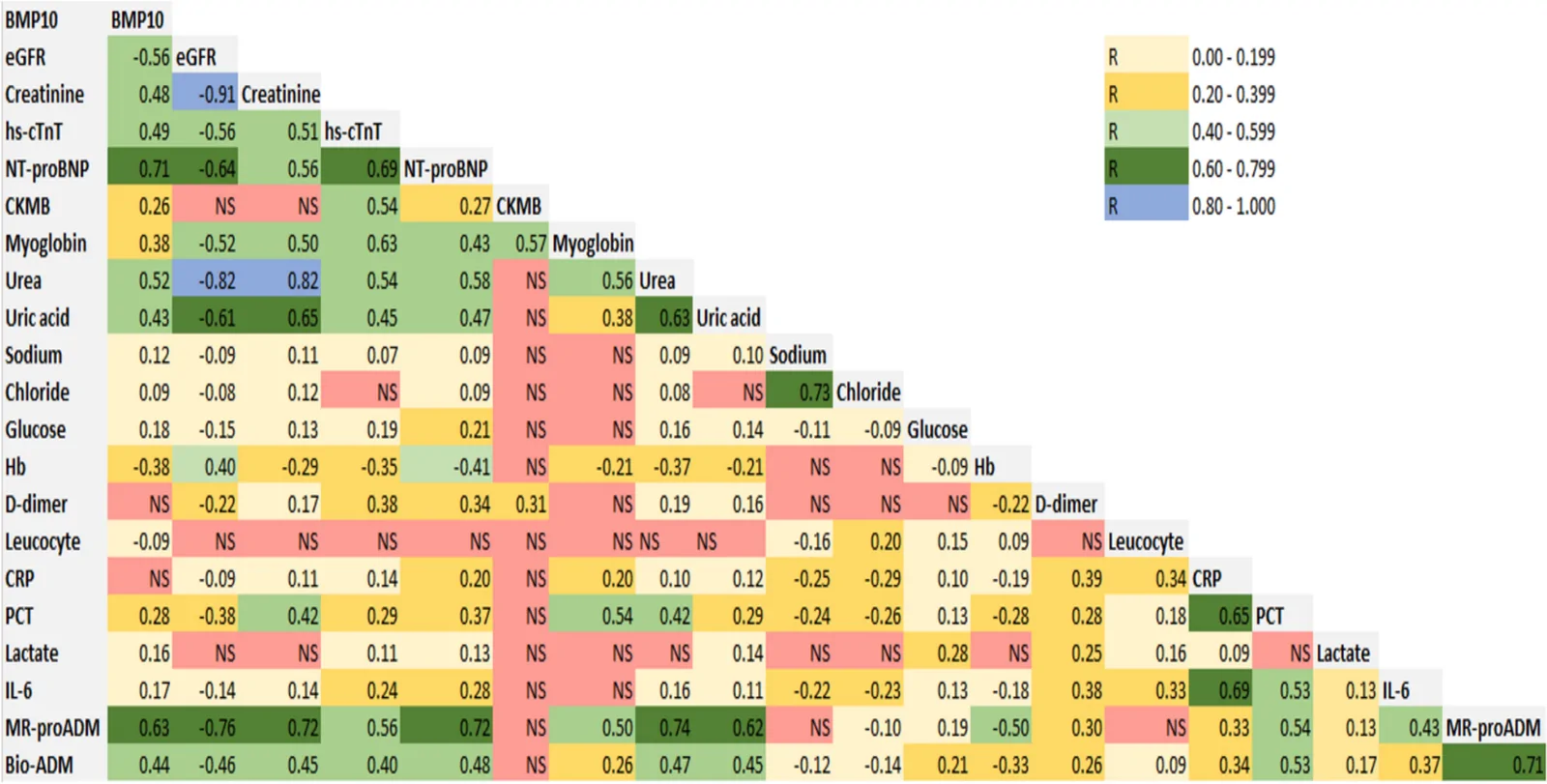
Spearmen’s correlation coefficients between different blood biomarkers. *Bio-ADM* bioactive adrenomedullin, *BMP10* Bone Morphogenetic Protein 10, *CKMB* creatinine kinase isoenzyme-MB, *CRP* C-reactive protein, *eGFR* estimated glomerular filtration rate, *Hb* hemoglobin, *hs-cTnT* high-sensitivity cardiac troponin T, *IL-6* interleukin-6, *MR-proADM* mid-regional proadrenomedullin, *NS* not significant, *NT-proBNP* N-terminal pro-B-type natriuretic peptide, *PCT* procalcitonin

Correlation between different blood biomarkers is depicted in Fig. 5. Plasma BMP10 concentration showed a strong correlation between NT-proBNP (0.71, *p* < 0.001) and MR-proADM (0.63, *p* < 0.001).

### Plasma BMP10 and cardiac morphology and function

There was a weak correlation between plasma BMP10 level and parameters of cardiac morphology and function, particularly left atrial size (Supplemental Table 2).

### BMP10 and AHF triggers

**Fig. 6:**
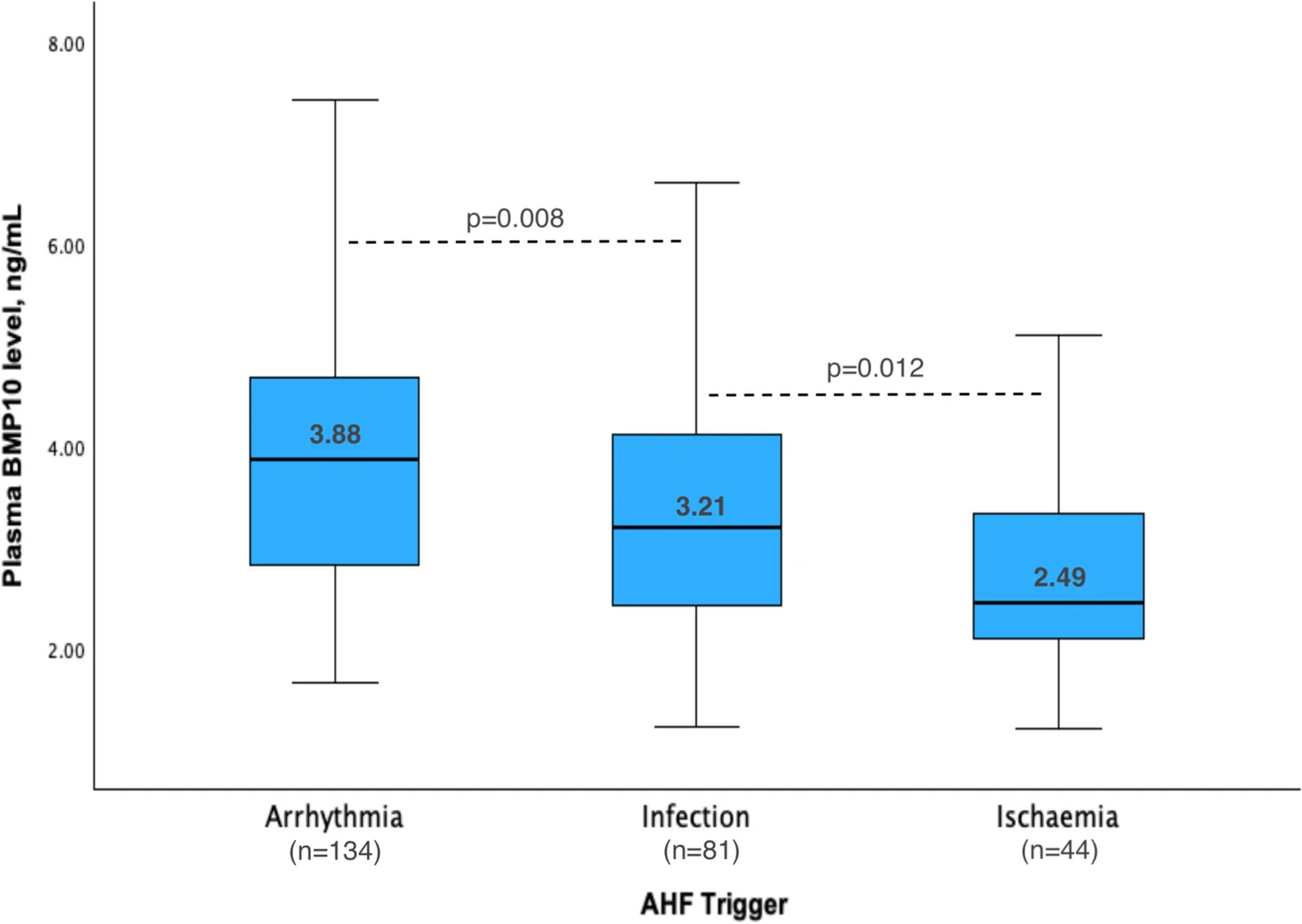
Plasma BMP10 concentration among patients with different triggers of AHF. Plasma BMP10 concentrations among patients with different triggers of AHF: arrhythmia 3.88 (2.84–4.72) ng/mL, infection 3.21 (2.43–4.19) ng/mL, and ischemia 2.49 (2.09–3.39) ng/mL. *AHF* acute heart failure, *BMP10* Bone Morphogenetic Protein 10

BMP10 concentration differed according to the trigger of AHF, with the highest values observed in AHF triggered by tachyarrhythmia 3.88 (2.84–4.72) ng/mL (Fig. 6).

We further tested the ability of BMP10, NT-proBNP, and hs-cTnT to identify AHF cases triggered by specific triggers.

#### Arrhythmia

AUC for BMP10 to identify arrhythmia-triggered AHF profile from the entire acute dyspnea cohort was the highest (0.78, 95% CI 0.75–0.82), followed by NT-proBNP (AUC 0.73, 95% CI 0.69–0.76, p for comparison 0.021) and hs-cTnT (0.56, 95% CI 0.52–0.61, *p* for comparison < 0.001; Supplemental Fig. 2A).

#### Myocardial ischemia

Hs-cTnT performed best for myocardial ischemia-related AHF (AUC 0.85, 95% CI 0.80–0.90), followed by NT-proBNP (0.71, 95% CI 0.65–0.77), and BMP10 (0.57, 95% CI 0.50–0.63). The combination of BMP10 and NT-proBNP did not outperform NT-proBNP alone (AUC 0.69, 95% CI 0.63–0.75, *p* for comparison = 0.556, Supplemental Fig. 2B).

#### Infection

For infection, the AUCs were as follow: 0.77, 95% CI 0.73–0.81 for NT-proBNP, 0.68, 95% CI 0.64–0.73 for BMP10, and 0.67, 95% CI 0.62–0.71 for hs-cTnT. The combination of BMP10 and NT-proBNP again did not outperform NT-proBNP alone (AUC 0.76, 95% CI 0.72–0.80, *p* for comparison = 0.766, Supplemental Fig. 2C).

### Prognostic implications

The prognostic properties of BMP10 were investigated in 933 acute dyspnea patients and in a subgroup of 502 patients with adjudicated AHF. Within the acute dyspnea cohort, 29% (267 out of 933) of the patients died within the 720-day follow-up period (Supplemental Table 3). Among the AHF patients, the mortality rate was even higher, with 38% (188 out of 502) of the patients dying within the 720-day follow-up period (Supplemental Table 4).

### Prognostic role of BMP10 in acute dyspnea

**Fig. 7:**
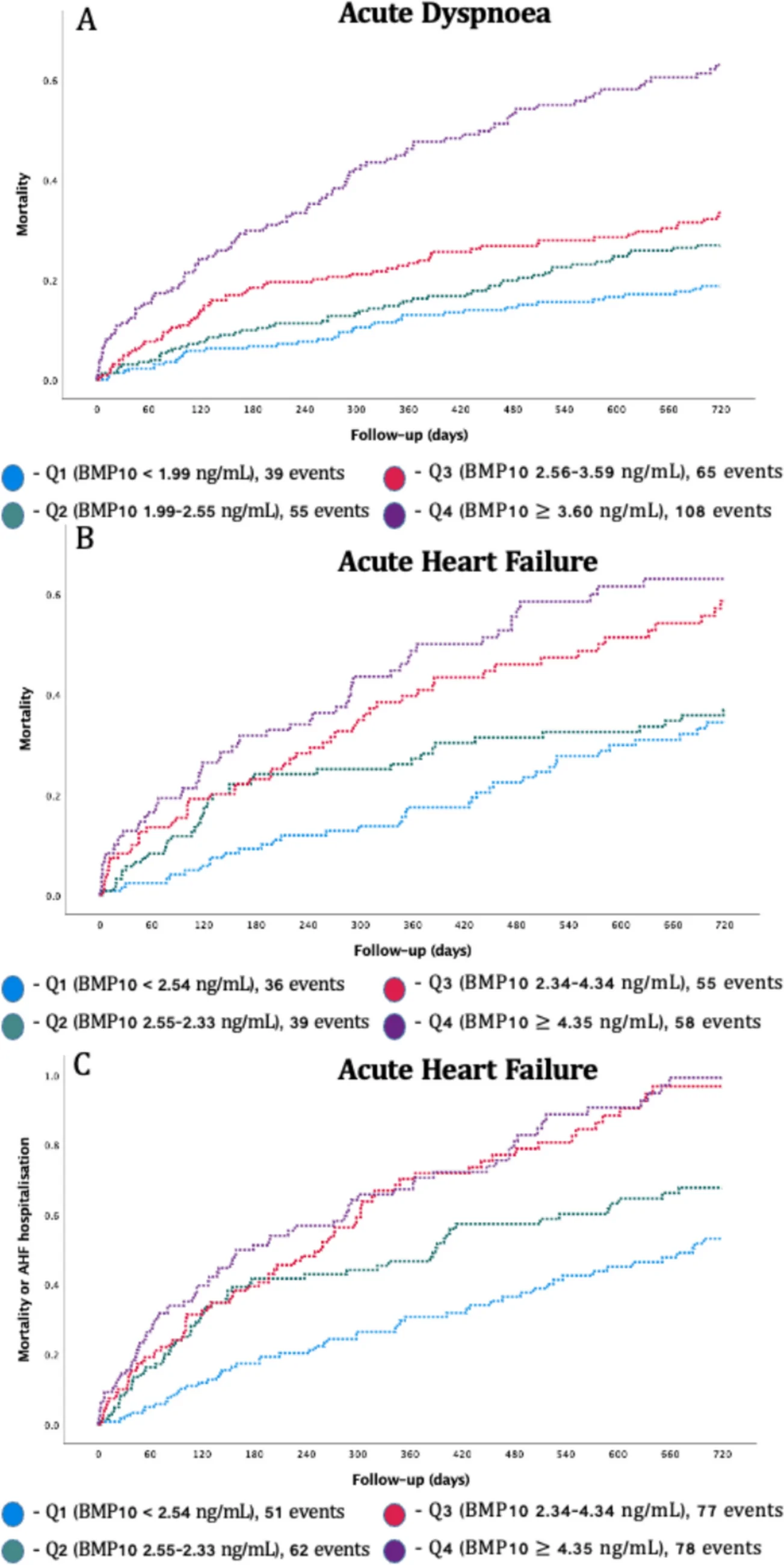
Hazard curves of mortality in the overall group of patients with acute dyspnea (**A**) and the subgroup of acute heart failure (**B**) stratified by quartiles of plasma BMP10 levels at presentation. In the subgroup of acute heart failure, also the composite of mortality or rehospitalization for acute heart failure (**C**) is shown. *BMP10* Bone Morphogenetic Protein 10, *Q* quartile

Plasma BMP10 concentration was a strong predictor of mortality in patients with acute dyspnea, with patients in the fourth quartile having nearly four-times the mortality of patients in the first quartile (Fig. 7A). As a continuous variable in univariable analysis, the HR was 1.22 (95% CI 1.17–1.28, *p* < 0.001). However, after multivariable adjustment including NT-proBNP, BMP10 lost its predictive value (HR 1.01, 95% CI 0.94–1.09), *p* = 0.745, (Supplemental Table 5).

### Prognostic role of BMP10 in AHF

Plasma BMP10 concentration was a strong predictor of mortality in patients with adjudicated AHF, with patients in the fourth quartile having about three times the mortality of patients in the first quartile (Fig. 7B). As a continuous variable in univariable analysis, the HR was 1.16 (95% CI 1.09–1.23, *p* < 0.001). However, after multivariable adjustment, BMP10 lost its predictive value (HR 1.02, 95% CI 0.94–1.10, *p* = 0.711). Similar findings were found for the composite of death or AHF rehospitalization.

### The fraction of new information

The added value of BMP10 for predicting the risk of death in acute dyspnea, death in AHF, and death or rehospitalization for AHF was 0.2%, 0.2%, and 2%, respectively. The risk was estimated for a follow-up time of 720 days.

## Discussion

To the best of our knowledge, this was the first study assessing the diagnostic and prognostic utility of BMP10, a novel atrial-specific biomarker, in patients presenting with acute dyspnea to the ED. We report six major findings.

First, the plasma concentration of BMP10 was significantly higher in patients adjudicated to have AHF versus those with other causes of acute dyspnea and had high diagnostic accuracy for AHF (AUC 0.85). The diagnostic accuracy of BMP10 for AHF was even higher versus hs-cTnT (0.79), another cardiac-specific biomarker, but lower versus NT-proBNP, the gold standard in the diagnosis of AHF. This provides further support for the evolving concept of BMP10 as an atrial-specific biomarker [8, 16–19] as increased atrial pressure and increased atrial size are hallmarks of AHF [27]. The inferiority of BMP10 versus NT-proBNP in the diagnosis of AHF may at least in part be explained by inclusion bias as according to current guidelines and in order to maximize the accuracy of the central adjudication, it had to include BNP or NT-proBNP concentrations, while it was blinded to BMP10. Second, in contrast to our hypothesis, the combination of BMP10 with NT-proBNP did not significantly increase the diagnostic accuracy provided by NT-proBNP alone (AUC 0.92 versus 0.91, *p* = ns). This important observation may be due to the high correlation between BMP10 and NT-proBNP (r = 0.71), indicating a large overlap in the pathophysiological triggers of the release of BMP10 and NT-proBNP. In addition, it suggests that the presence and the extent of hemodynamic stress affecting the ventricles and the atria in most AHF phenotypes seen in contemporary clinical practice in Europe are similar. Different findings may emerge in countries with a higher prevalence of severe mitral or tricuspid stenosis, which would result in disproportional hemodynamic stress to the atria. Third, in the particularly challenging subgroup of patients with a history of atrial fibrillation, BMP10 provided comparable diagnostic accuracy versus NT-proBNP for AHF. However, also in this subgroup, the combination of BMP10 with NT-proBNP did not significantly increase the diagnostic accuracy provided by NT-proBNP alone. Fourth, BMP10 concentrations differed according to AHF phenotype and were the highest in patients with cardiogenic shock and patients with isolated acute right heart failure. The later finding is supported by observations made in an invasive study suggesting pre-capillary pulmonary hemodynamic as the dominant trigger of BMP10 release [15]. Indeed, in a very recent study, we have been able to demonstrate that BMP10 is very closely related to pre-capillary pulmonary hemodynamic, but not with pulmonary arterial wedge pressure [15]. Fifth, BMP10 provided the highest AUC among the three investigated cardiac biomarkers to identify tachyarrhythmia as the trigger of AHF. This is well in line with the association between atrial fibrillation and BMP10 observed in prior studies [16–19]. Six, BMP10 was a powerful predictor of premature death during both short- and long-term follow-ups. The prognostic accuracy overall was comparable to that of NT-proBNP, but not additive to multivariate models including NT-proBNP. Similar to the diagnostic indication, the strong correlation between BMP10 and NT-proBNP, and the excellent performance of NT-proBNP likely contributed to the lack of incremental prognostic value on top of NT-proBNP.

These findings corroborate and extend recent experimental and clinical studies characterizing BMP10 as a heart-restricted right atrial-specific circulating biomarker [8, 9, 28, 29], with strong prognostic capability for adverse outcomes including stroke, death, and recurrence in patients with atrial fibrillation. For instance, a very large cohort study including 16,053 patients with atrial fibrillation found a strong, independent, and consistent relationship between plasma BMP10 concentration and the risk of ischemic stroke [17]. Importantly, this association was present irrespective of NT-proBNP concentration and treatment with oral anticoagulants [17]. Although in patients with atrial fibrillation BMP10 concentration was independently associated with heart failure hospitalizations, we did not find this independent prognostic value in our cohort. This discrepancy can be explained by prominent differences in study cohorts with our population being older and presenting with much higher NT-proBNP plasma levels, different triggers of BMP10 release, as well as adjustment methodology. Although statistically significant, the improvement in the C-index among atrial fibrillation patients was very low (≤ 0.005), potentially revealing limited clinical significance.

Over the past two decades, natriuretic peptides have been integral in the diagnostic and risk assessment pathways of acute dyspnea and AHF [3, 30, 31]. These markers, indicative of myocardial hemodynamic stress, are released in response to increased intracardial pressure, a fundamental pathophysiological aspect of AHF, thus making them ideal for diagnostic purposes. Yet, their diagnostic accuracy is considerably diminished in atrial fibrillation patients [6, 7], who constitute approximately one-third of heart failure cases [32]. It is a matter of debate to what extent this observation is related to a true failure of BNP or NT-proBNP, or merely uncertainty and inconsistency in the diagnosis of AHF in the presence of atrial fibrillation [27]. Perhaps at least in part, the failure of BMP10 to increase the diagnostic accuracy of NT-proBNP in patients with atrial fibrillation was due to these uncertainties and the following limitation and added noise in the central adjudication. Until more precise diagnostic tools become available, the clinical evaluation of dyspnoic patients with atrial fibrillation should not be limited to NT-proBNP measurements. It should encompass a comprehensive range of diagnostic techniques, clinical history, and other clinical findings, including weight gain, rales, jugular venous distension, leg edema, chest X-ray, and CT scans to detect and differentiate pulmonary embolism, pulmonary venous congestion, infiltrates, malignancies, and other conditions. Advanced ultrasound techniques for assessing heart, lungs, and great vessels should also be included in the evaluation of patients with uncertain diagnosis.

This study has several limitations. First, our findings regarding diagnostics and prognosis are specific to patients presenting with acute dyspnea to the ED and cannot be generalized to patients presenting with less severe symptoms to primary care. This research question requires future studies. Second, despite the strong methodology used for the central adjudication of the final diagnosis, we still might have misclassified a small proportion of patient, potentially reducing the power of this study to demonstrate incremental diagnostic value for BM10. Third, to maximize the accuracy of central adjudication, measurements of BNP or NT-proBNP were available for the cardiologists during the adjudication process, creating an unavoidable inclusion bias in favor of NT-proBNP. Accordingly, the real difference in diagnostic accuracy between BMP10 and NT-proBNP may be smaller than found in this study. Fourth, the study did not include patients with terminal renal failure on chronic dialysis. Thus, we cannot comment on the possible value of BMP10 in this vulnerable patient population. Fifth, the present study included patients with acute dyspnea and a subgroup with AHF. Although these populations overlap with other cardiovascular pathologies, such as acute coronary syndromes, pulmonary embolism, and pulmonary hypertension, our data were not powered to provide more in-depth insights into these subgroups, which require further investigation in appropriately designed cohorts.

In conclusion, in patients presenting with acute dyspnea to the ED, BMP10 had a high diagnostic accuracy for AHF, as well as high prognostic accuracy for death and AHF rehospitalization. However, it did not provide relevant incremental value to the current gold standard NT-proBNP. Future studies might wish to focus on its possible role in identifying atrial fibrillation as the trigger of AHF.

### Clinical perspectives

The clinical implications of our findings seem to be the following. First, despite its impressive diagnostic and prognostic performance, the incremental value of BMP10 to the current gold standard NT-proBNP seemed very small or even absent in the established indications: the diagnosis of AHF and the prediction of death. Accordingly, further research on BMP10 is mandatory to better understand its pathophysiology including triggers and modifiers, and define its possible clinical sweet-spot, in which its use really could provide incremental value to patients and physicians. Second, as identification of the trigger of AHF is central for cause-specific treatment, BMP10 may have a role to identify atrial fibrillation as the primary trigger in patients who may present after spontaneous cardioversion to sinus rhythm to the ED, leaving the true trigger unidentified and missing targeted treatment opportunities. Our study shows BMP10’s effectiveness in identifying arrhythmia-triggered AHF, significantly surpassing NT-proBNP. While this is an exploratory finding, its potential clinical utility in providing decision support and tailored treatment options may be significant.

## Supplementary Information

Below is the link to the electronic supplementary material.

Supplementary file1 (DOCX 38 KB)

Supplementary file2 (PNG 163 KB)

Supplementary file3 (TIFF 531 KB)
