# Supplementary material for: Diagnostic and prognostic utility of bone morphogenetic protein 10 in acute dyspnea: a cohort study
Source: Clin Res Cardiol. 2024 Dec 11;115(11):1897–909. doi: 10.1007/s00392-024-02584-2 (PMC13658398; doi:10.1007/s00392-024-02584-2)
Supplement: Supplementary file 1 [file 392_2024_2584_MOESM1_ESM.docx]

Supplemental Tables

| Supplemental Table 1. Plasma BMP10 concentration in different diagnostic categories of non-AHF causes of acute dyspnoea | | | | |
| --- | --- | --- | --- | --- |
| Causes of dyspnoea |  | **Yes** | **No** | ***p*-value** |
| COPD/Asthma | n (%) | 138 (32%) | 293 (68%) |  |
|  | BMP10 (ng/mL) | 2.05 (1.80-2.35) | 2.03 (1.72-2.49) | 0.782 |
| Pulmonary embolism | n (%) | 45 (10%) | 386 (90%) |  |
|  | BMP10 (ng/mL) | 2.11 (1.84-2.69) | 2.03 (1.74-2.43) | 0.052 |
| Pneumonia | n (%) | 94 (22%) | 337 (78%) |  |
|  | BMP10 (ng/mL) | 2.17 (1.68-2.61) | 2.02 (1.77-2.41) | 0.302 |
| Hyperventilation | n (%) | 37 (9%) | 394 (91%) |  |
|  | BMP10 (ng/mL) | 1.90 (1.67-2.30) | 2.06 (1.76-2.46) | 0.086 |
| Obesity | n (%) | 7 (2%) | 424 (98%) |  |
|  | BMP10 (ng/mL) | 1.69 (1.62-2.29) | 2.05 (1.76-2.46) | 0.337 |
| Bronchitis | n (%) | 17 (4%) | 414 (96%) |  |
|  | BMP10 (ng/mL) | 2.08 (1.74-2.61) | 2.04 (1.75-2.45) | 0.382 |

| Supplemental Table 2. Correlation between parameters of cardiac morphology and function, and plasma BMP10 level | | |
| --- | --- | --- |
| Left ventricular end diastolic diameter (cm) | Spearman`s rank (rs) | 0.21 |
|  | *p*-value | <0.001 |
|  | n | 376 |
| Left ventricular end systolic diameter (cm) | Spearman`s rank (rs) | 0.24 |
|  | *p*-value | <0.001 |
|  | n | 377 |
| Left ventricular mass (g) | Spearman`s rank (rs) | 0.20 |
|  | *p*-value | <0.001 |
|  | n | 376 |
| Left ventricular mass index (g/m2) | Spearman`s rank (rs) | 0.27 |
|  | *p*-value | <0.001 |
|  | n | 370 |
| Left ventricular ejection fraction (%) | Spearman`s rank (rs) | -0.36 |
|  | *p*-value | <0.001 |
|  | n | 453 |
| Left atrium (cm) | Spearman`s rank (rs) | 0.37 |
|  | *p*-value | <0.001 |
|  | n | 383 |
| E/A-ratio | Spearman`s rank (rs) | 0.24 |
|  | *p*-value | <0.001 |
|  | n | 196 |

| Supplemental Table 3. Baseline characteristics of acute dyspnoea patients stratified by the 720-day survival status | | | |
| --- | --- | --- | --- |
|  | **Dead at 720 days**  **(n-267)** | **Alive at 720 days**  **(n-666)** | **P-value** |
| Demographics | | | |
| Age (years) | 81 (73-86) | 72 (60-81) | <0.001 |
| Female sex | 104 (39%) | 312 (47%) | 0.029 |
| Weight (kg) | 70 (59-82) | 78 (65-90) | <0.001 |
| BMI (kg/m^2^) | 24.9 (21.6-28.4) | 26.4 (23.4-31.0) | <0.001 |
| Clinical parameters at presentation | | | |
| SBP (mmHg) | 129 (112-146) | 138 (122-155) | <0.001 |
| Heart rate (bpm) | 86 (74-101) | 92 (75-108) | 0.008 |
| Respiratory rate (breaths/minute) | 22 (18-28) | 22 (17-28) | 0.226 |
| Pulse oximetry (%) | 95 (91-98) | 96 (93-98) | 0.018 |
| Temperature (°C) | 37.1 (36.7-37.6) | 37.3 (36.9-37.7) | 0.003 |
| Medical history | | | |
| Hypertension | 216 (81%) | 451 (68%) | <0.001 |
| Dyslipidaemia | 147 (55%) | 301 (45%) | 0.007 |
| CAD | 128 (48%) | 214 (32%) | <0.001 |
| Prior MI | 92 (35%) | 110 (17%) | <0.001 |
| Prior AHF | 136 (51%) | 183 (28%) | <0.001 |
| Atrial fibrillation | 100 (38%) | 184 (28%) | 0.004 |
| CKD | 128 (48%) | 142 (21%) | <0.001 |
| PAD | 43 (16%) | 63 (10%) | 0.006 |
| Stroke | 45 (17%) | 69 (10%) | 0.008 |
| COPD/Asthma | 95 (36%) | 227 (34%) | 0.703 |
| Medication at presentation | | | |
| ACE inhibitors | 108 (41%) | 190 (29%) | <0.001 |
| ARBs | 53 (20%) | 155 (23%) | 0.296 |
| Beta-blockers | 159 (60%) | 295 (44%) | <0.001 |
| Aldosterone antagonists | 38 (14%) | 44 (7%) | <0.001 |
| CCB | 57 (21%) | 119 (18%) | 0.229 |
| Digoxin | 10 (4%) | 15 (2%) | 0.260 |
| Diuretics | 192 (72%) | 309 (47%) | <0.001 |
| Echocardiography | | | |
| LVEF (%) | 45 (30-60) | 55 (40-60) | 0.001 |
| Laboratory parameters | | | |
| Haemoglobin (g/L) | 121 (109-134) | 137 (124-149) | <0.001 |
| Haematocrit (%) | 36 (32-40) | 39 (36-43) | <0.001 |
| Sodium (mmol/L) | 138 (136-141) | 139 (136-141) | 0.865 |
| Chloride (mmol/L) | 100 (96-103) | 101 (97-104) | 0.046 |
| Potassium (mmol/L) | 4.2 (3.8-4.7) | 4.0 (3.7-4.3) | <0.001 |
| Creatinine (µmol/L) | 106 (79-157) | 85 (68-111) | <0.001 |
| eGFR (ml/min/1.73 m^2^) | 50 (33-74) | 71 (48-90) | <0.001 |
| Urea (mmol/L) | 10.1 (7.2-16.1) | 7.0 (5.0-10.0) | <0.001 |
| Albumin (g/L) | 33 (31-33) | 36 (33-39) | <0.001 |
| NT-proBNP (ng/L) | 4126 (1282-10570) | 890 (190-3871) | <0.001 |
| Hs-cTnT (ng/L) | 43 (24-78) | 19 (9-36) | <0.001 |
| BMP10 (ng/mL) | 3.20 (2.29-4.29) | 2.39 (1.91-3.28) | <0.001 |

*ACE: angiotensin-converting enzyme, AHF: acute heart failure, ARB: angiotensin II receptor blocker, BMI: body mass index, CAD: coronary artery disease, BMP10: Bone Morphogenetic Protein 10, CCB: calcium channel blocker, CKD: chronic kidney disease, COPD: chronic obstructive pulmonary disease, eGFR: estimated glomerular filtration rate, Hs-cTnT: high-sensitivity cardiac troponin T, LVEF: left ventricular ejection fraction, MI: myocardial infarction, NT-proBNP: N-terminal pro-B-type natriuretic peptide, PAD: peripheral artery disease, SBP: systolic blood pressure*

| Supplemental Table 4. Baseline characteristics of acute heart failure patients stratified by the 720-day survival status | | | |
| --- | --- | --- | --- |
|  | **Dead at 720 days**  **(n-188)** | **Alive at 720 days**  **(n-314)** | **P-value** |
| Demographics | | | |
| Age (years) | 83 (77-87) | 78 (67-83) | <0.001 |
| Female sex | 78 (42%) | 132 (42%) | 0.926 |
| Weight (kg) | 70 (60-84) | 80 (69-93) | <0.001 |
| BMI (kg/m^2^) | 24.9 (21.8-28.7) | 27.0 (24.0-31.3) | <0.001 |
| Clinical parameters at presentation | | | |
| SBP (mmHg) | 127 (111-144) | 141 (122-158) | <0.001 |
| Heart rate (bpm) | 84 (73-100) | 90 (71-109) | 0.092 |
| Respiratory rate (breaths/minute) | 22 (18-28) | 22 (18-28) | 0.680 |
| Pulse oximetry (%) | 95 (90-98) | 96 (94-98) | 0.043 |
| Temperature (°C) | 37.0 (36.5-37.6) | 37.2 (36.7-37.6) | 0.156 |
| Medical history | | | |
| Hypertension | 158 (84%) | 266 (85%) | 0.799 |
| Dyslipidaemia | 114 (61%) | 175 (56%) | 0.305 |
| CAD | 103 (55%) | 148 (47%) | 0.117 |
| Prior MI | 75 (40%) | 79 (25%) | <0.001 |
| Prior AHF | 122 (65%) | 153 (49%) | <0.001 |
| Atrial fibrillation | 87 (46%) | 157 (50%) | 0.461 |
| CKD | 104 (55%) | 102 (33%) | <0.001 |
| PAD | 38 (20%) | 36 (12%) | 0.009 |
| Stroke | 33 (18%) | 44 (14%) | 0.307 |
| COPD/Asthma | 56 (30%) | 69 (22%) | 0.055 |
| Medication at presentation | | | |
| ACE inhibitors | 86 (46%) | 118 (38%) | 0.091 |
| ARBs | 38 (20%) | 96 (31%) | 0.012 |
| Beta-blockers | 122 (65%) | 203 (65%) | 1.000 |
| Aldosterone antagonists | 31 (17%) | 34 (11%) | 0.076 |
| CCB | 40 (21%) | 71 (23%) | 0.740 |
| Digoxin | 8 (4%) | 12 (4%) | 0.818 |
| Diuretics | 147 (78%) | 209 (67%) | 0.008 |
| Echocardiography | | | |
| LVEF (%) | 42 (28-55) | 50 (35-58) | 0.907 |
| Laboratory parameters | | | |
| Haemoglobin (g/L) | 119 (108-132) | 131 (117-142) | <0.001 |
| Haematocrit (%) | 36 (32-39) | 38 (34-42) | <0.001 |
| Sodium (mmol/L) | 139 (137-141) | 139 (137-142) | 0.137 |
| Chloride (mmol/L) | 100 (96-104) | 102 (98-104) | 0.014 |
| Potassium (mmol/L) | 4.3 (3.9-4.7) | 4.1 (3.8-4.5) | 0.002 |
| Creatinine (µmol/L) | 119 (88-173) | 95 (79-129) | <0.001 |
| eGFR (ml/min/1.73 m^2^) | 42 (27-63) | 59 (39-77) | <0.001 |
| Urea (mmol/L) | 11.2 (8.4-17.9) | 8.7 (6.2-12.0) | <0.001 |
| Albumin (g/L) | 33 (31-36) | 35 (32-38) | <0.001 |
| NT-proBNP (ng/L) | 6847 (3390-16142) | 3632 (1788-7117) | <0.001 |
| Hs-cTnT (ng/L) | 54 (33-95) | 29 (18-59) | <0.001 |
| BMP10 (ng/mL) | 3.76 (2.73-4.60) | 3.17 (2.44-4.13) | <0.001 |

*ACE: angiotensin-converting enzyme, AHF: acute heart failure, ARB: angiotensin II receptor blocker, BMI: body mass index, CAD: coronary artery disease, BMP10: Bone Morphogenetic Protein 10, CCB: calcium channel blocker, CKD: chronic kidney disease, COPD: chronic obstructive pulmonary disease, eGFR: estimated glomerular filtration rate, Hs-cTnT: high-sensitivity cardiac troponin T, LVEF: left ventricular ejection fraction, MI: myocardial infarction, NT-proBNP: N-terminal pro-B-type natriuretic peptide, PAD: peripheral artery disease, SBP: systolic blood pressure*

| Supplemental Table 5. Multivariable Cox proportional hazards models | | | |
| --- | --- | --- | --- |
| ACUTE DYSPNOEA | | | |
| 720-day mortality (n = 864) | | | |
| Variable | HR | 95% CI | p |
| BMP10 (ng/mL) | 1.01 | 0.94-1.09 | 0.745 |
| Age (year) | 1.05 | 1.03-1.06 | <0.001 |
| Heart rate (bpm) | 0.998 | 0.993-1.004 | 0.501 |
| Urea (mmol/l) | 1.04 | 1.02-1.06 | <0.001 |
| NYHA class | 1.28 | 1.05-1.57 | 0.015 |
| NT-proBNP (ng/L) | 1.00 | 1.00-1.00 | <0.001 |
| SBP (mmHg) | 0.991 | 0.986-0.997 | 0.002 |
| Heart murmur | 1.05 | 0.80-1.39 | 0.715 |
| ACUTE HEART FAILURE | | | |
| 720-day mortality (n = 486) | | | |
| Variable | HR | 95% CI | p |
| BMP10 (ng/mL) | 1.02 | 0.94-1.10 | 0.711 |
| Age (year) | 1.04 | 1.02-1.06 | <0.001 |
| Beta blocker at presentation | 1.00 | 0.73-1.37 | 0.992 |
| Urea (mmol/l) | 1.028 | 1.006-1.049 | 0.011 |
| NTproBNP (ng/L) | 1.00 | 1.00-1.00 | <0.001 |
| Hb (g/L) | 0.991 | 0.984-0.997 | 0.015 |
| 720-day mortality or heart failure hospitalisation (n = 482) | | | |
| Variable | HR | 95% CI | p |
| BMP10 (ng/mL) | 1.03 | 0.96-1.11 | 0.347 |
| Age (year) | 1.02 | 1.01-1.03 | 0.002 |
| Beta blocker at presentation | 0.976 | 0.74-1.28 | 0.863 |
| Urea (mmol/l) | 1.03 | 1.01-1.05 | 0.003 |
| NT-proBNP (ng/L) | 1.00 | 1.00-1.00 | <0.001 |
| Hb (g/L) | 0.995 | 0.989-1.002 | 0.143 |
| Oedema | 0.99 | 0.76-1.29 | 0.926 |
| History of HF | 1.52 | 1.16-1.99 | 0.002 |
| Sodium (mmol/l) | 0.98 | 0.96-1.01 | 0.131 |

| Supplemental Table 6. Multivariable Cox proportional hazards models in patients with eGFR <60 ml/min/1.73 m^2^ | | | |
| --- | --- | --- | --- |
| ACUTE DYSPNOEA | | | |
| 720-day mortality (n = 383) | | | |
| Variable | HR | 95% CI | p |
| BMP10 (ng/mL) | 1.00 | 0.92-1.09 | 0.965 |
| Age (year) | 1.05 | 1.02-1.07 | <0.001 |
| Heart rate (bpm) | 0.997 | 0.990-1.059 | 0.380 |
| Urea (mmol/l) | 1.04 | 1.01-1.06 | 0.002 |
| NYHA class | 1.14 | 0.88-1.47 | 0.327 |
| NT-proBNP (ng/L) | 1.00 | 1.00-1.00 | <0.001 |
| SBP (mmHg) | 0.994 | 0.988-1.00 | 0.101 |
| Heart murmur | 1.13 | 0.81-1.58 | 0.461 |
| ACUTE HEART FAILURE | | | |
| 720-day mortality (n = 286) | | | |
| Variable | HR | 95% CI | p |
| BMP10 (ng/mL) | 1.00 | 0.92-1.10 | 0.948 |
| Age (year) | 1.04 | 1.02-1.07 | <0.001 |
| Beta blocker at presentation | 0.92 | 0.63-1.35 | 0.673 |
| Urea (mmol/l) | 1.032 | 1.007-1.058 | 0.011 |
| NTproBNP (ng/L) | 1.00 | 1.00-1.00 | <0.001 |
| Hb (g/L) | 0.992 | 0.984-1.001 | 0.070 |
| 720-day mortality or heart failure hospitalisation (n = 284) | | | |
| Variable | HR | 95% CI | p |
| BMP10 (ng/mL) | 1.02 | 0.94-1.10 | 0.668 |
| Age (year) | 1.03 | 1.01-1.05 | 0.001 |
| Beta blocker at presentation | 0.83 | 0.60-1.15 | 0.267 |
| Urea (mmol/l) | 1.04 | 1.03-1.06 | 0.001 |
| NT-proBNP (ng/L) | 1.00 | 1.00-1.00 | 0.006 |
| Hb (g/L) | 0.997 | 0.990-1.005 | 0.455 |
| Oedema | 0.97 | 0.70-1.234 | 0.832 |
| History of HF | 1.48 | 1.06-2.07 | 0.021 |
| Sodium (mmol/l) | 0.97 | 0.94-1.00 | 0.069 |
